# The effect of sulindac on redox homeostasis and apoptosis-related proteins in melanotic and amelanotic cells

**DOI:** 10.1007/s43440-023-00493-1

**Published:** 2023-05-17

**Authors:** Maciej Miliński, Monika Staś, Jakub Rok, Artur Beberok, Dorota Wrześniok

**Affiliations:** 1 Faculty of Chemistry, University of Opole, Oleska 48, 45-052 Opole, Poland; 2 Department of Pharmaceutical Chemistry, School of Pharmacy With the Division of Laboratory Medicine in Sosnowiec, Medical University of Silesia in Katowice, Jagiellońska 4, 41-200 Sosnowiec, Poland

**Keywords:** Sulindac, Dacarbazine, Melanotic melanoma, Amelanotic melanoma, Cancer, p53/Bax/Bcl-2 proteins

## Abstract

**Background:**

Non-steroidal anti-inflammatory drugs have been shown to inhibit the development of induced neoplasms. Our previous research demonstrated that the cytotoxicity of sulindac against melanoma cells is comparable to dacarbazine, the drug used in chemotherapy. The aim of this study was to investigate the mechanism of sulindac cytotoxicity on COLO 829 and C32 cell lines.

**Methods:**

The influence of sundilac on the activity of selected enzymes of the antioxidant system (superoxide dismutase (SOD), catalase (CAT), and glutathione peroxidase (GPx)) and the content of hydrogen peroxide as well as the level of proteins initiating (p53, Bax) and inhibiting (Bcl-2) apoptosis were measured in melanoma cells.

**Results:**

In melanotic melanoma cells, sulindac increased the activity of SOD and the content of H_2_O_2_ but decreased the activity of CAT and GPx. The level of p53 and Bax proteins rose but the content of Bcl-2 protein was lowered. Similar results were observed for dacarbazine. In amelanotic melanoma cells, sulindac did not cause an increase in the activity of measured enzymes or any significant changes in the level of apoptotic proteins.

**Conclusion:**

The cytotoxic effect of sulindac in the COLO 829 cell line is connected to disturbed redox homeostasis by changing the activity of SOD, CAT, GPx, and level of H_2_O_2_. Sulindac also induces apoptosis by changing the ratio of the pro-apoptotic/anti-apoptotic protein. The presented studies indicate the possibility of developing target therapy against melanotic melanoma using sulindac.

**Graphical abstract:**

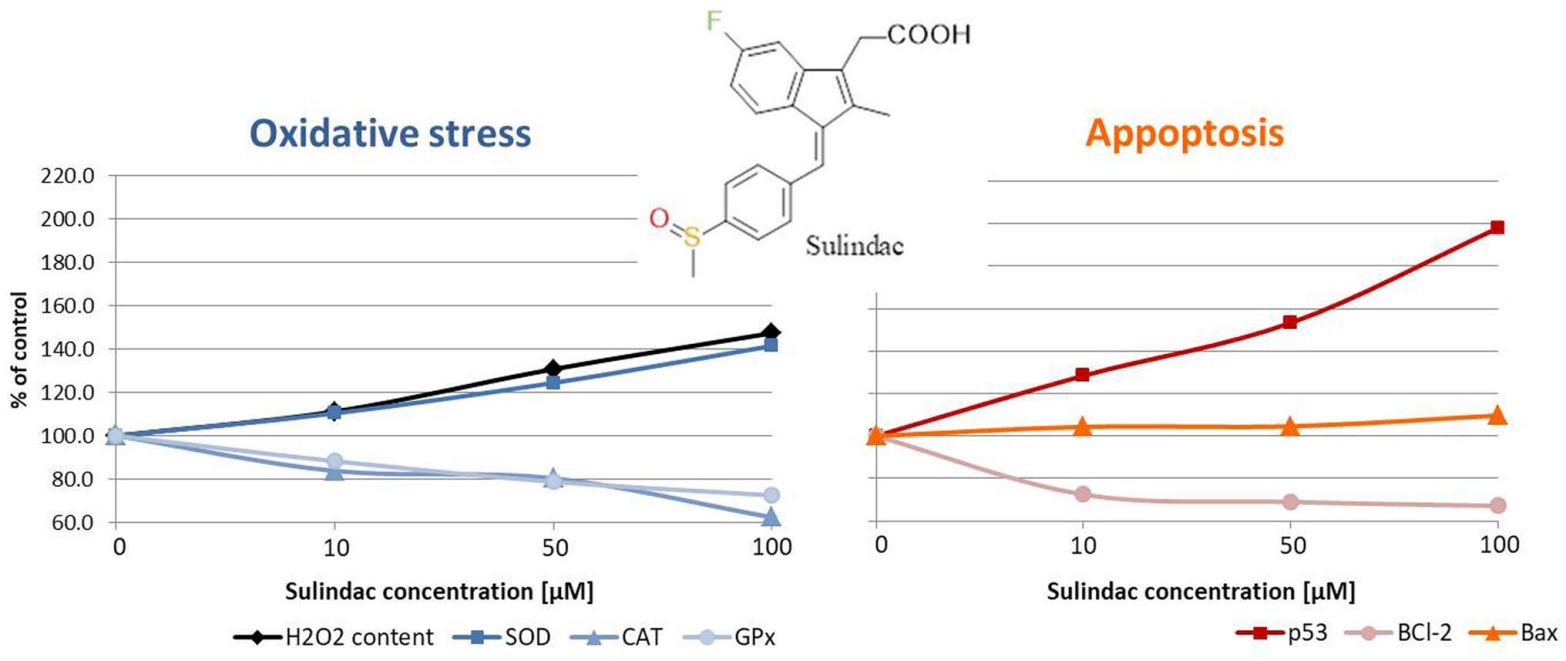

**Supplementary Information:**

The online version contains supplementary material available at 10.1007/s43440-023-00493-1.

## Introduction

DNA damage may cause uncontrolled growth of cells leading to various types of cancer. Exposure to direct sunlight, especially ultraviolet light (UVB and UVA) induces the development of melanoma cells. A malignant neoplasm matures as a result of the neoplastic transformation of pigmented nevi or arises de novo in unchanged skin, mucous membranes, or the eyeball. Due to the aggressive course of the disease and the tendency to the occurrence of early and numerous metastases via the lymphatic system and bloodstream, melanoma is considered the most malignant type of skin and mucosa cancer [1, 2]. There are two basic types of melanoma, melanotic and amelanotic, of which the latter is not yet well understood. It is characterized by low melanin content and less cell differentiation, as well as a faster growth rate, greater malignancy, and worse prognosis than in the case of the melanotic variant [3, 4].

**Fig. 1:**
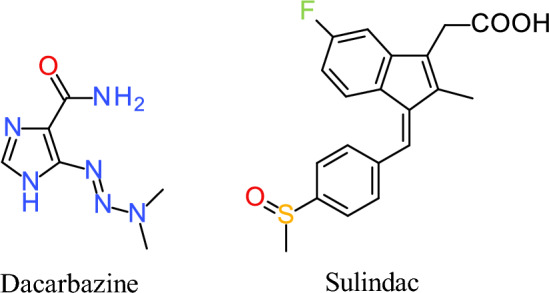
The chemical structure of the studied drugs

The basic method of treating melanoma is a surgical removal of the lesion. The scope of the procedure depends on the stage and location of the lesion as well as the clinical condition of the lymph nodes. Other methods, including chemotherapy, immunotherapy, targeted therapy, or radiation (phototherapy) are also often utilized [5]. Since 1975, dacarbazine has been used in chemotherapy mainly in stage IV metastatic malignant melanoma. Dacarbazine (Fig. 1) is a hydrophobic, phase-unspecific, pharmacologically inactive alkylating agent belonging to triazene. It has a short half-life and serious side effects [5]. Dacarbazine is considered the reference drug for evaluating new anti-melanoma compounds [6, 7].

The experimental and epidemiological studies conducted in recent years showed that non-steroidal anti-inflammatory drugs (NSAIDs) have anti-cancer properties [8–12]. The first indications of possible non-standard applications of COX inhibitors appeared in the 1980s. In experimental studies on animals, Pollard et al. [13] showed that chronic administration of indomethacin in laboratory rats with chemically induced colorectal cancer has a protective effect. The animals treated with indomethacin for several weeks had a significantly lower number and smaller size of tumors. The mechanism of NSAIDs’ anti-tumor activity is related, *inter alia*, to their inhibitory effect on tumor angiogenesis as well as the induction of apoptosis in neoplastic cells. Sulindac, acetylsalicylic acid, celecoxib, and indomethacin are NSAIDs often studied in the context of anti-tumor activity [5, 9, 14–16].

Sulindac (Fig. 1) is an indene analog of indomethacin, substituted indene acetic acid with a fluorine atom and sulfoxide group. It is used in the treatment of rheumatoid arthritis, ankylosing spondylitis, osteoarthritis, gout, and tendinitis [17]. In 1983, Waddell and Loughry [18] used sulindac in four patients with familial polyposis of the large intestine, achieving complete regression of the lesions after 1 year of treatment. Similar effects were observed after the 5 year treatment period. The key role of sulindac in the aforementioned clinical effect was confirmed by the fact that discontinuation of therapy was associated with the recurrence of the disease [19]. Over the years, the effectiveness of sulindac against various cancer lines has been proven in many experimental studies [9, 15, 20, 21]. Studies confirmed the inhibitory effect of sulindac on the NF-κB transcription factor in colon and breast cancer cells, leading to disturbance of the growth processes of neoplastic cells [15, 22]. Incubation of squamous cell carcinoma with sulindac induced apoptosis and inhibited tumor growth [23]. It was also reported that this drug downregulates Sp. transcription factors and Sp-regulated pro-oncogenic gene products [20] leading to the inhibition of cell cycle progression and induction of the apoptosis process caused by the accumulation of reactive oxygen species (ROS) in the cells. This is associated with the opening of mitochondrial channels, the release of cytochrome C, induction of p38 MAPK kinase, accumulation of pro-apoptotic proteins: p53, Bac, and Bax, and reduction in the level of the anti-apoptotic protein Bcl-2 [24]. The induction of pro-apoptotic NAG-1 protein under the influence of sulindac has also been demonstrated in studies carried out on ovarian cancer cells [25].

In our previous study [26], we demonstrated that the survival rate of melanotic (COLO 829) melanoma cells cultured in the presence of sulindac decreased with increasing drug concentration, and the effect was comparable to the reference drug, dacarbazine. Both tested drugs reduced the survival rate of amelanotic (C32) melanoma cells to a much lesser extent. For sulindac, 100 μM concentration exhibits the highest observed cytotoxic effect on COLO829 cells (decrease of cell viability to 53% of control). For C32 cells, sulindac did not affect viability.

This study’s aim is to investigate the cytotoxicity mechanism of sulindac on malignant melanoma cells. To determine the antioxidant potential and apoptosis induction of sulindac, the H_2_O_2_ content and activity of superoxide dismutase (SOD), catalase (CAT), and glutathione peroxide (GPx) were measured, as well as the content of apoptotic proteins: p53, Bcl-2, and Bax. The evaluation of the enzyme activities and protein levels in melanotic and amelanotic melanoma cells incubated with sulindac was compared with the reference drug, dacarbazine.

## Materials and methods

Sulindac (S8139), dacarbazine (D2390), and amphotericin B (0.25 mg/ml) (A2942) were purchased from Sigma-Aldrich Inc. (USA). Growth medium RPMI 1604 with l-glutamate (L0495) and growth medium DMEM with l-glutamine (L0102) and glucose, as well as penicillin (P0018), streptomycin (S6501), and trypsin solutions (L0909) were purchased from Cytogen (Poland). Cell Proliferation Reagent I—WST-1 (11 644 807 001) was obtained from F. Hoffmann-La Roche AG.

### Cell culture

Two malignant melanoma cell lines: melanotic (COLO 829) and amelanotic (C32) were purchased from ATCC. The quantitative composition of the basic culture medium for cells of the COLO829 line was: 500 ml of RMPI 1640, 55 ml of inactivated fetal bovine serum, 1 ml of solution of penicillin (10,000 U/ml) and streptomycin (10 mg/ml), 1 ml of amphotericin B solution (0.25 mg/ml). Quantitative composition of the basic culture medium for cells of the C32 line was: 500 ml of DMEM, 55 ml of inactivated fetal bovine serum, 1 ml of solution of penicillin (10,000 U/ml) and streptomycin (10 mg/ml), 1 ml of amphotericin B solution (0.25 mg/ml).

### Measurements of SOD, CAT, and GPx activity in cancer cells

Superoxide dismutase activity in melanoma cells was determined using the SOD Assay Kit (706,002; Cayman Chemical). CAT activity was determined using the Catalase Assay Kit (707,002; Cayman Chemical). GPx activity was determined using the Glutathione Peroxidase Assay Kit (703,102; Cayman Chemical). Each test were performed five times with 1 × 10^5^ cells/trial. COLO 829 melanotic melanoma cells and C32 amelanotic melanoma cells were treated with 10 µM, 50 µM, and 100 µM drug concentrations.

### Determination of p53, Bcl-2, Bax proteins and H2O2 content in melanoma cells

Proteins’ content was determined in cell lysates by the modified Lowry method (sodium citrate was used), which uses a sensitive reaction of peptide bonds and aromatic amino acids with the phenolic reagent of Folin–Ciocalteu [27, 28].

Hydrogen peroxide content was determined using the OxiSelect™ Hydrogen Peroxide Assay Kit (STA-844), purchased from Cell Biolabs. Proteins p53, Bcl-2, and Bax in melanoma cells were determined by enzyme immunoassay using the following assays: human p53 ELISA (RAF082R; BioVendor), human Bcl-2 ELISA (RAF005R; BioVendor), Bax human ELISA kit (502,006,852; Enzo). Each test was performed three or four times with 2.5 × 10^5^ cells/trial.

### Statistical analysis

Mean values and standard deviations (SD) were calculated. After checking whether the distribution of results in individual groups is consistent with the normal distribution and whether the variances of comparable groups meet the assumption of homogeneity (Brown–Forsythe test), a one-way ANOVA and Dunnett’s test were used. The results were considered statistically significant at *p* < 0.05. GraphPadPrism 6.01 software was used to calculate the variability values.

## Results

### The effect of sulindac and dacarbazine on the antioxidant potential of melanotic and amelanotic melanoma cells

The effect of sulindac and dacarbazine on the antioxidant potential of melanoma cells was measured by the activity of three enzymes: superoxide dismutase (SOD), catalase (CAT), and glutathione peroxidase (GPx) as well as by the content of hydrogen peroxide in the presence of the drugs in various concentrations. The measurements of enzyme activity were performed by a spectrophotometric method using cell lysates obtained after 24 h incubation of melanoma cells in the presence of the tested drugs.

### Superoxide dismutase (SOD) activity

**Fig. 2:**
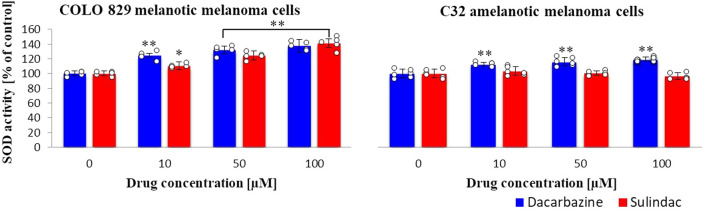
Superoxide dismutase (SOD) activity in COLO 829 melanotic and C32 amelanotic melanoma cells grown in the presence of dacarbazine and sulindac; SOD Assay Kit. One-way ANOVA followed by Dunnet’s test. The data are shown as a percentage of SOD activity to control, mean ± SD, *n* = 5, *p < 0.05, **p < 0.01. Circles represent individual data points. More details are included in Tables 1S and 2S in the Supplementary materials

Superoxide dismutase activity increased in melanoma cells in presence of sulindac proportional to the concentration in comparison to the control (Fig. 2). In melanotic melanoma cells incubated with 100 µM sulindac solution, the enzyme activity increased by 41.4 ± 8.7%, similar to the reference drug, dacarbazine, for which the activity increases by 37 ± 5.7%. In the case of amelanotic melanoma cells incubated in sulindac solution, the SOD activity did not change, whereas dacarbazine caused higher SOD activity than in the control sample. The effect of dacarbazine on amelanotic cells is much smaller than on melanotic melanoma cells. (sulindac: COLO 829 F_3,16_ = 54, *p* < 0.0001; C32 F_3,20_ = 2.1, *p* = 0.1377; dacarbazine: COLO 829 *F*_3,16_ = 50, *p* < 0.0001).

### Catalase (CAT) activity

**Fig. 3:**
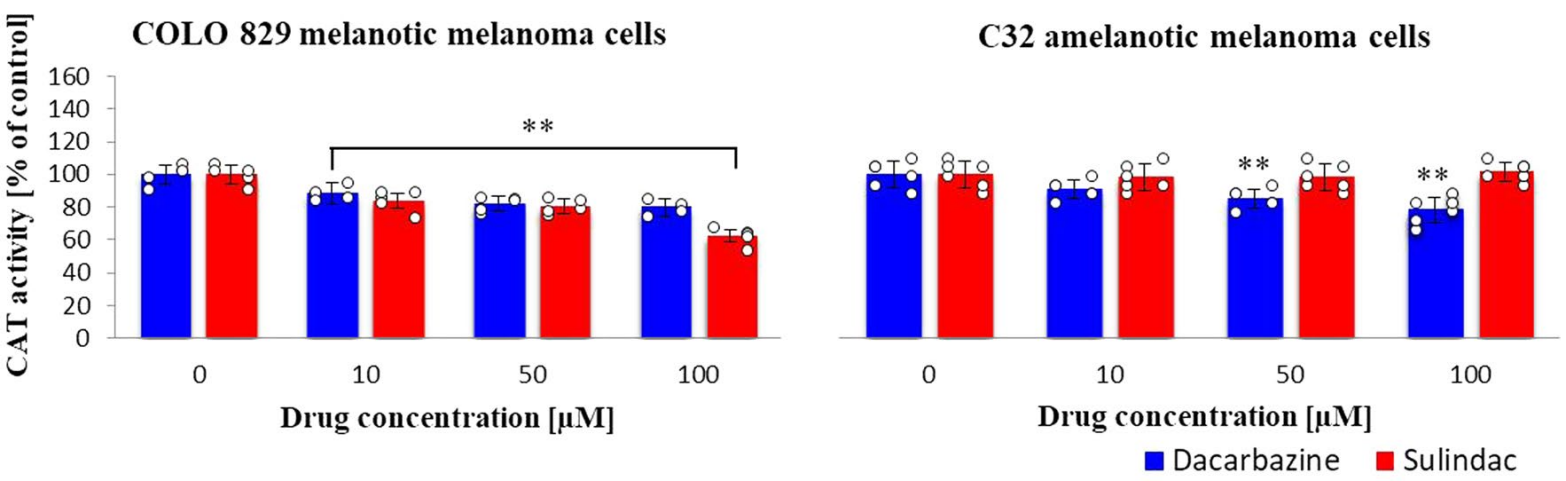
Catalase (CAT) activity in COLO 829 melanotic and C32 amelanotic melanoma cells grown in the presence of dacarbazine and sulindac; Catalase Assay Kit. One-way ANOVA followed by Dunnet’s test. The data are shown as a percentage of catalase activity to control, mean ± SD, n = 3, *p < 0.05, **p < 0.01. Circles represent individual data points. More details are included in Tables 3S and 4S in the Supplementary materials

In the presence of sulindac, the catalase activity decreased proportionally to the concentration in melanotic melanoma cells (Fig. 3) but no changes were observed for amelanotic cells. In contrast to dacarbazine, which caused a lowering of CAT activity in both types of melanoma cells. (sulindac: COLO 829 *F*_3,16_ = 38, *p* < 0.0001; C32 *F*_3,20_ = 0.3, *p* = 0.8219; dacarbazine: COLO 829 *F*_3,16_ = 18, *p* < 0.0001; C32 *F*_3,20_ = 10, *p* = 0.0002.)

### Glutathione peroxidase (GPx) activity

**Fig. 4:**
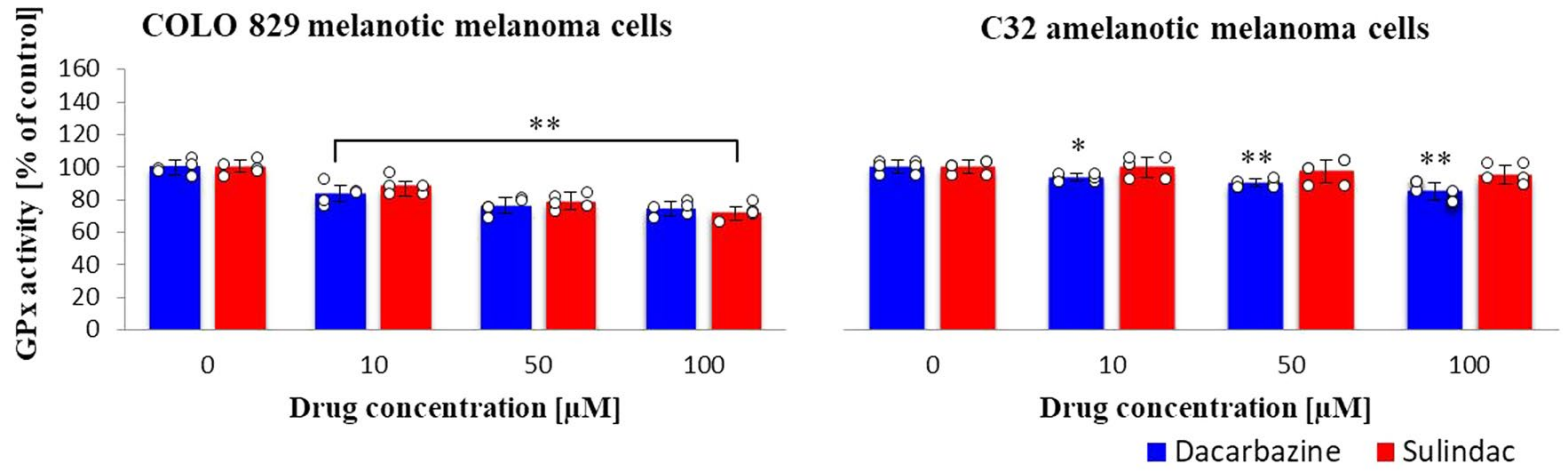
Glutathione peroxidase (GPx) activity in COLO 829 melanotic and C32 amelanotic melanoma cells grown in the presence of dacarbazine and sulindac expressed; Glutathione Peroxidase Assay Kit. One-way ANOVA followed by Dunnet’s test. The data are shown as a percentage of glutathione peroxide activity to control, mean ± SD, *n* = 3, *p < 0.05, **p < 0.01. Circles represent individual data points. More details are included in Tables 5S and 6S in the Supplementary materials

Sulindac and dacarbazine caused a similar effect of glutathione peroxide activity in melanotic melanoma cells. Sulindac did not affect the GPx activity of glutathione peroxidase in amelanotic melanoma cells, while dacarbazine caused a decrease in its activity compared to the control (Fig. 4). In amelanotic melanoma cells, sulindac at all tested concentrations did not cause statistically significant changes, while with increasing dacarbazine concentration, GPx activity in the tested cells decreased; however, the decrease was smaller than for melanotic melanoma cells. (sulindac: COLO 829 *F*_3,16_ = 33, *p* < 0.0001; C32 *F*_3,20_ = 0.97, *p* = 0.4258; dacarbazine: COLO 829 *F*_3,16_ = 29, *p* < 0.0001; C32 *F*_3,20_ = 17, *p* < 0.0001.)

### Hydrogen peroxide content

**Fig. 5:**
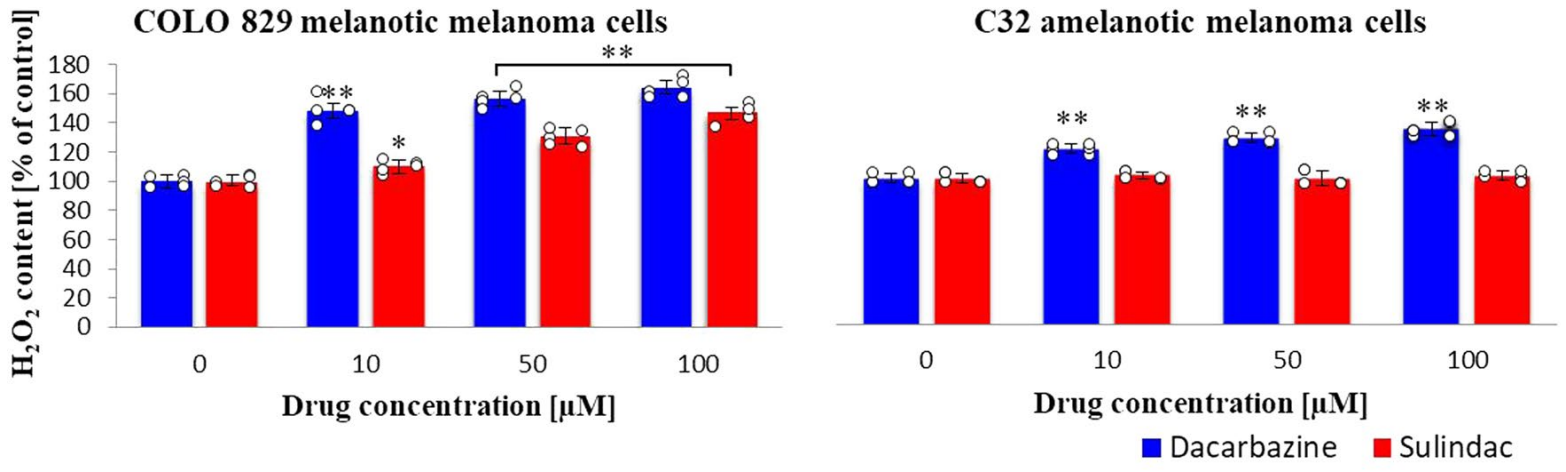
Hydrogen peroxide content in COLO 829 melanotic and C32 amelanotic melanoma cells grown in the presence of dacarbazine and sulindac; OxiSelectTM Hydrogen Peroxide Assay Kit. One-way ANOVA followed by Dunnet's test. The data are shown as a percentage of H_2_O_2_ content to control, mean ± SD, *n* = 3, *p < 0.05, **p < 0.01. Circles represent individual data points. More details are included in Tables 7S and 8S in the Supplementary materials

The impact of sulindac and dacarbazine on the content of H_2_O_2_ in melanoma cells was also measured (Fig. 5). Hydrogen peroxide levels in melanotic melanoma cells in the presence of sulindac increased compared to the control sample. In the case of incubation of cells with dacarbazine solutions, even the lowest used concentration caused a statistically significant increase in the content of H_2_O_2_ higher than incubation with sulindac. In amelanotic melanoma cells, sulindac did not influence the level of hydrogen peroxide. On the other hand, dacarbazine caused smaller changes than in melanotic melanoma cells. (sulindac: COLO 829 *F*_3,20_ = 116, *p* < 0.0001; C32 *F*_3,20_ = 0.55, *p* = 0.6524; dacarbazine: COLO 829 *F*_3,16_ = 71, *p* < 0.0001; C32 *F*_3,20_ = 96, *p* < 0.0001.)

### Effect of sulindac on the expression of apoptotic proteins in melanoma cells

The influence of sulindac and dacarbazine on their ability to initiate the process of apoptosis in malignant melanoma cells was measured by the content of p53, Bcl-2, and Bax proteins in the presence of the drugs in various concentrations. The protein content level in melanotic and amelanotic melanoma cells was determined in cell lysates after 24 h incubation with the drug.

### Protein p53 content

**Fig. 6:**
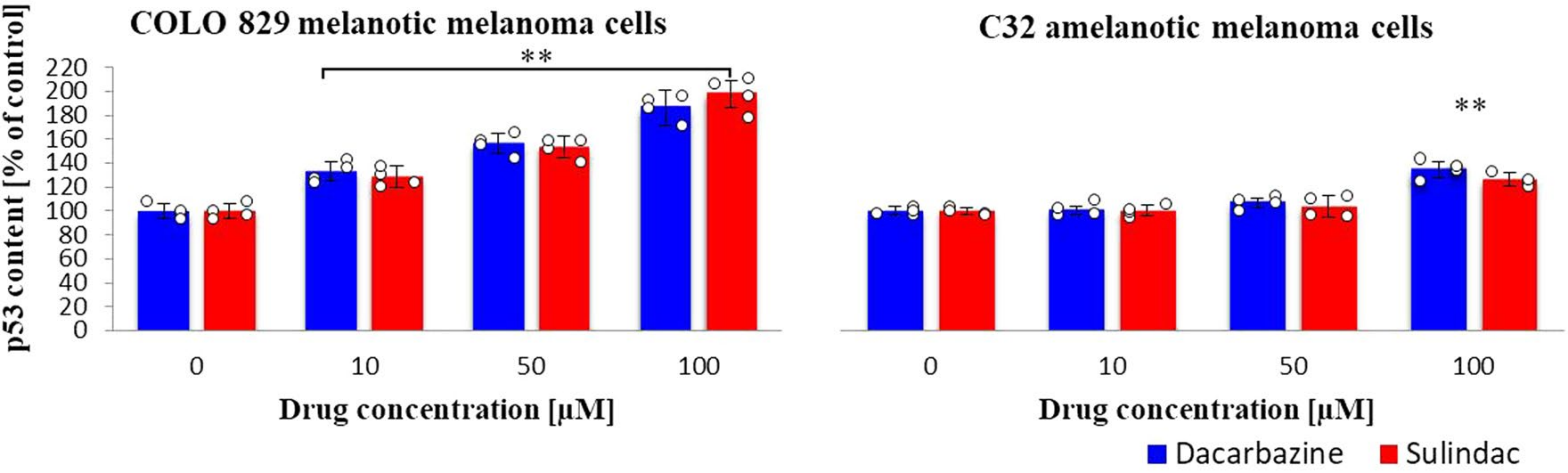
p53 protein content in COLO 829 melanotic and C32 amelanotic melanoma cells grown in the presence of dacarbazine and sulindac; Human p53 ELISA (Biovendor) Kit. One-way ANOVA followed by Dunnet’s test. The data are shown as a percentage of p53 protein content to control, mean ± SD, *n* = 4, *p < 0.05, **p < 0.01. Circles represent individual data points. More details are included in Tables 9S and 10S in supporting information.

Incubation of melanotic melanoma cells with various concentrations of sulindac increased the p53 protein content compared to the control sample (Fig. 6). A similar dependence was seen in the case of dacarbazine; increasing the drug dose increased the p53 protein content. On the other hand, the influence of the studied drugs on the p53 protein content in C32 cells was much smaller and the effect was only observed for the highest concentration of 100 μM. (sulindac: COLO 829 *F*_3,12_ = 71, *p* < 0.0001; C32 *F*_3,12_ = 17, *p* = 0.0001; dacarbazine: COLO 829 *F*_3,12_ = 66, *p* < 0.0001; C32 *F*_3,20_ = 33, *p* < 0.0001.)

### Bcl-2 protein content

**Fig. 7:**
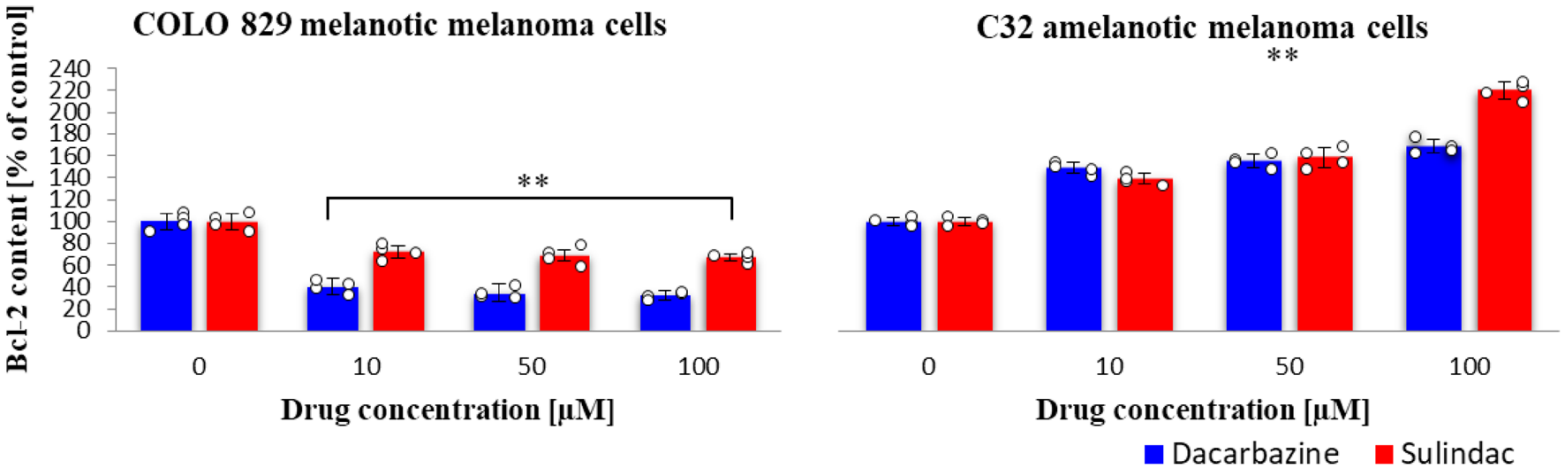
Bcl-2 protein content in COLO 829 melanotic and C32 amelanotic melanoma cells grown in the presence of dacarbazine and sulindac; Human Bcl-2 ELISA (Biovendor) Kit. One-way ANOVA followed by Dunnet’s test. The data are shown as a percentage of Bcl-2 protein content to control, mean ± SD, *n* = 4, *p < 0.05, **p < 0.01. Circles represent individual data points. More details are included in Tables 11S and 12S in the Supplementary materials

The Bcl-2 protein content was affected by sulindac in COLO 829 cells decreasing its amount in the sample (Fig. 7). A much larger decrease was noted for the incubation of these cells with dacarbazine. In amelanotic cells, the trend was the opposite, the content of Bcl-2 protein increased in the cells for both drugs. (sulindac: COLO 829 *F*_3,12_ = 20, *p* < 0.0001; C32 *F*_3,12_ = 221, *p* < 0.0001; dacarbazine: COLO 829 *F*_3,12_ = 134, *p* < 0.0001; C32 *F*_3,12_ = 123, *p* < 0.0001).

### Bax protein content

**Fig. 8:**
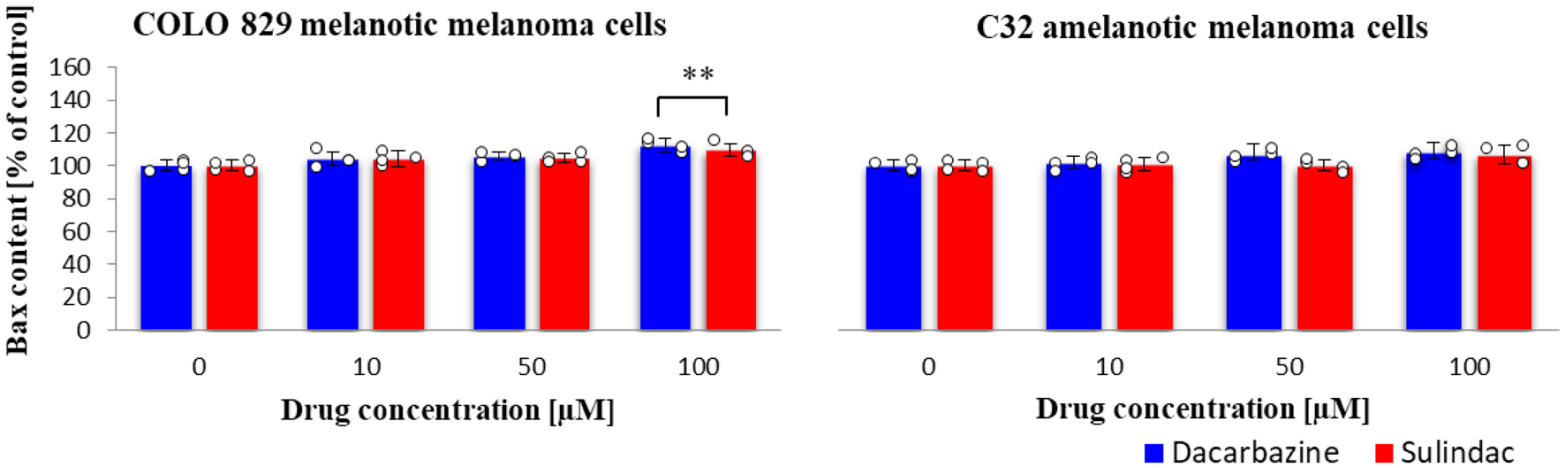
Bax protein content in COLO 829 melanotic and C32 amelanotic melanoma cells grown in the presence of dacarbazine and sulindac; Bax (human) ELISA Kit (Enzo). One-way ANOVA followed by Dunnet's test. The data are shown as a percentage of Bax protein content to control, mean ± SD, *n* = 4, *p < 0.05, **p < 0.01. Circles represent individual data points. More details are included in Tables 13S and 14S in the Supplementary materials

Both tested drugs slightly affected the Bax protein content in melanotic and amelanotic cells (Fig. 8). Only in the case of the highest concentration (100 µM), significant increase was observed compared to the control sample. Incubation of amelanotic cells with sulindac and dacarbazine caused a similar effect. (sulindac: COLO 829 *F*_3,12_ = 4.9, *p* = 0.0183; C32 *F*_3,12_ = 2.3, *p* = 0.1278; dacarbazine: COLO 829 *F*_3,12_ = 8.5, *p* = 0.0027; C32 *F*_3,12_ = 5.8, *p* = 0.0107.)

## Discussion

There is a limited selection of cytostatics in systemic therapy; additionally, melanoma cells are highly resistant to most known treatments. The prognosis of patients diagnosed with melanoma is lowered, due to high resistance to the induction of apoptosis [29]. Therefore, constant efforts to develop new methods of treatment should be made. According to numerous studies, NSAIDs exhibit clinically significant anti-cancer properties, among others is sulindac [8–12].

In this article, we attempt to determine the mechanism of the cytotoxic mechanism of sulindac. For this purpose, the influence of the tested drug, in various concentrations, on the activity of antioxidant enzymes (SOD, CAT, and GPx) and the amount of hydrogen peroxide, as well as the content of proteins stimulating (p53, Bax) and inhibiting (Bcl-2) the apoptosis process in COLO 829 melanotic and C32 amelanotic cell lines were measured. The anti-melanoma properties of sulindac were compared with the effects of the reference drug, dacarbazine. Dacarbazine is the most commonly used drug in clinical practice during chemotherapy. The rate of response to this drug is approximately 20% [30].

Our studies showed that in COLO 829 melanotic cell line, the activity of SOD and H_2_O_2_ content increased with increasing concentration of sulindac as opposed to the activity of CAT and GPx. The content of p53 protein increased, unlike the level of Bcl-2 and Bax proteins. A stronger effect was observed when cells were incubated with dacarbazine. In C32 amelanotic cell lines, the activity of measured enzymes and the content of H_2_O_2_ as well as the level of selected proteins significantly changed neither in presence of sulindac nor dacarbazine.

The research was inspired by our previous studies, where we proved that sulindac decreases the survival rate of melanotic (COLO 829) melanoma cells and to a much lesser extent, amelanotic (C32) melanoma cells. Sulindac was much more cytotoxic to the melanotic melanoma cells of the COLO 829 line than to the amelanotic melanoma cells of the C32 line. The survival of melanoma cells was decreasing proportionally to the concentrations of sulindac. It should also be noticed that sulindac had a slightly higher cytotoxicity to melanotic melanoma cells than the reference drug, dacarbazine [26].

The cytotoxicity mechanism of sulindac was investigated by the activity of antioxidant enzymes—SOD, CAT, and GPx. Those enzymes regulate the reactive oxygen species content in the cell, changes in their activity may cause cell damage, induce apoptosis, and reduce cell proliferation [31]. The activity of antioxidant enzymes has a large influence on the treatment of many diseases, including cancer. Verm et al. [32] investigated the sulindac influence on the activity of antioxidant enzymes in rat lung cells affected by fibrosis caused by bleomycin administration. In the cells incubated with sulindac, the activity of antioxidant enzymes increases enhancing the antioxidant defence of the cells against bleomycin.

The cytotoxicity is also related to apoptosis induction. Therefore, the content of three proteins was measured—p53, Bcl-2, and Bax. The p53 protein is a transcription factor responsible for the regulation of the cell cycle and, in particular, for the activation of DNA repair and the induction of apoptosis [33]. It is inactive in the cell until the appearance of various types of stress factors, such as heat, oxidative stress, or pathological conditions associated with the genetic instability of cells and further with the development of cancer. Activation of p53 involves the phosphorylation of one of several specific Ser/Thr residues within the N-terminal or C-terminal domain of the protein due to the presence of several kinase enzymes that interact with p53 in response to a physiological imbalance in the body. When DNA repair fails, p53 initiates cell apoptosis by increasing Bax expression and mitochondrial release of cytochrome C and AIF (apoptosis-inducing factor), while reducing the concentration of Bcl-2 responsible for inhibiting the secretion of these factors [34].

Raisova et al. [35] proved that the mutual quantitative ratio of Bax and Bcl-2 proteins determines the initiation of the apoptosis process in melanoma cells of the M186 and M221 lines. For the ratio < 1 of Bax/Bcl-2 in the analyzed cells, the proliferative processes had the advantage over apoptosis. On the other hand, the increase in the Bax/Bcl-2 ratio above the > 1 value was associated with the increased sensitivity of the tested cells to the induction of the apoptosis process. In addition, the cited studies have shown that the p53 protein by attaching to the Bax protein promotes its oligomerization, which intensifies the process of apoptosis, and the occurrence of mutations within p53 reduces the intensity of this process in melanoma cells. Similar conclusions were drawn from studies by Faião-Flores et al. [36] who assessed the effect of the curcumin analog DM1 on the survival of murine melanoma cells of the B16F10 line. They recognized that the resistance of melanoma to apoptosis is one of its most important properties and that the effectiveness of anti-cancer therapy depends on the possibility of its suppression. Using the DM1 compound, scientists were able to obtain a Bax/Bcl-2 ratio of 1.1, and when DM1 was used simultaneously with dacarbazine − 1.26. Comparing these results with those obtained in this study, it can be seen that the Bax/Bcl-2 ratio obtained as a result of sulindac incubation with COLO 829 cells is much higher − 1.64 (for the highest concentration used). In turn, the Bax/Bcl-2 ratio obtained for the amelanotic line was 0.49 for sulindac and dacarbazine was 0.64. The ratio decreases with increasing p53 protein content, which confirms that the p53 protein is the basic inhibitor of the proliferation of damaged cells and the multiplication of DNA errors, and the ratio of Bax to Bcl-2 ultimately determines their survival or death.

The increased expression of Bcl-2, and more precisely the unfavorable ratio of the pro-apoptotic Bax protein to the anti-apoptotic Bcl-2 protein, determines the high resistance of this tumor to chemotherapy. Therefore, in our studies, to evaluate the effect of sulindac on the content of pro- and anti-apoptotic proteins in malignant melanoma cells, the changes in the ratio of pro- and anti-apoptotic proteins from the Bcl-2 family were observed.

The obtained results for the amelanotic line confirmed the proposed mechanism. The slight cytotoxic effect of sulindac concerning C32 cells is a consequence of the production of a large amount of Bcl-2 protein in response to the action of the drug, inhibiting the initiation of the apoptosis process, and lack of influence on the level of the pro-apoptotic protein Bax. The reference drug, in contrast to sulindac, at the highest concentration used, slightly increases the content of Bax protein and to a lesser extent increases the content of Bcl-2 protein compared to the control sample. The differences in the content of pro- and anti-apoptotic proteins may explain the higher cytotoxicity of dacarbazine against C32 amelanotic melanoma cells in comparison to sulindac.

Summarizing the obtained results, sulindac cytotoxic activity against COLO 829 melanotic melanoma cells, comparable to the reference drug, dacarbazine, is related to the induction of oxidative stress in cancer cells and the stimulation of the apoptosis process. Results may suggest that apoptosis is initiated as a result of damage to cell structures caused by reactive oxygen species. Sulindac increases the level of pro-apoptotic proteins in COLO 829 melanotic melanoma cells in contrast to C32 cells. The obtained results are the basis for further research on sulindac's influence on melanotic melanoma cells, which may be used in the future to develop new targeted therapy against melanotic melanoma cells.

## Supplementary Information

Below is the link to the electronic supplementary material.

Supplementary file1 (PDF 583 KB)

## Data Availability

The data that support the findings of this study are available from the corresponding author upon reasonable request.

## Abbreviations

SOD: Superoxide dismutase,
CAT: Catalase
GPx: Glutathione peroxidase
H_2_O_2_: Hydrogen peroxide
COX: Cyclooxygenase
NSAIDs: Non-steroidal anti-inflammatory drugs
ROS: Reactive oxygen species
p53: Tumor protein p53 or transformation-related protein 53
Bax: Pro-apoptotic protein of the Bcl-2 family (Bcl-2-associated X protein)
Bcl-2: B-cell lymphoma 2 protein, member of pro- and anti-apoptotic proteins
