# Supplementary material for: The effect of sulindac on redox homeostasis and apoptosis-related proteins in melanotic and amelanotic cells
Source: Pharmacol Rep. 2023 May 17;75(4):995–1004. doi: 10.1007/s43440-023-00493-1 (PMC10374796; doi:10.1007/s43440-023-00493-1)
Supplement: Supplementary file 1 — Supplementary file1 (PDF 583 KB) [file 43440_2023_493_MOESM1_ESM.pdf]

Supporting information for

The effect of sulindac on redox homeostasis and apoptosis-related proteins in melanotic and amelanotic cells

Maciej Miliński[1]*, Monika Staś[1], Jakub Rok[2], Artur Beberok[2], Dorota Wrześniok[2]

[1] *Faculty of Chemistry, University of Opole, Oleska 48, Opole 45-052, Poland*

[2] *Department of Pharmaceutical Chemistry, School of Pharmacy with the Division of Laboratory Medicine in Sosnowiec, Medical University of Silesia in Katowice, Jagiellońska 4, 41-200, Sosnowiec, Poland*

Table 1S. Superoxide dismutase activity in COLO829 melanotic melanoma cells cultured in the presence of sulindac and dacarbazine.

Drug	Concentration [µM]	Average SOD activity		
		[U/ml] ± SD	[U/mg protein] ± SD	[%]
Sulindac	control	3.92 ± 0.13	1.74 ± 0.06	100.0 ± 3.3
	0.1	3.96 ± 0.18	1.80 ± 0.08	103.7 ± 4.6
	1.0	4.09 ± 0.19	1.84 ± 0.08	106.0 ± 4.8
	10	4.35 ± 0.11	1.92 ± 0.05	110.3 ± 2.7
	50	4.81 ± 0.20	2.16 ± 0.09	124.4 ± 5.1
	100	5.60 ± 0.34	2.46 ± 0.15	141.4 ± 8.7
Dacarbazine	control	3.92 ± 0.13	1.74 ± 0.06	100.0 ± 3.3
	0.1	4.08 ± 0.23	1.82 ± 0.10	104.8 ± 6.0
	1.0	4.27 ± 0.18	1.95 ± 0.08	112.3 ± 4.8
	10	4.85 ± 0.21	2.17 ± 0.09	124.7 ± 5.4
	50	5.06 ± 0.24	2.30 ± 0.11	132.0 ± 6.2
	100	5.40 ± 0.22	2.40 ± 0.10	137.9 ± 5.7

Table 2S. Superoxide dismutase activity in C32 amelanotic melanoma cells cultured in the presence of sulindac and dacarbazine.

Drug	Concentration [µM]	Average SOD activity		
		[U/ml] ± SD	[U/mg protein] ± SD	[%]
Sulindac	control	5.96 ± 0.33	3.08 ± 0.17	100.0 ± 5.6
	10	6.17 ± 0.35	3.18 ± 0.18	103.3 ± 5.9
	50	6.00 ± 0.18	3.09 ± 0.09	100.6 ± 3.0
	100	5.76 ± 0.25	2.97 ± 0.13	96.4 ± 4.2
Dacarbazine	control	5.96 ± 0.33	3.08 ± 0.17	100.0 ± 5.6
	10	6.73 ± 0.14	3.47 ± 0.07	112.7 ± 2.4
	50	6.92 ± 0.33	3.57 ± 0.17	116.0 ± 5.5
	100	7.10 ± 0.17	3.67 ± 0.09	119.2 ± 2.9

Table 3S. Catalase activity in COLO829 melanotic melanoma cells cultured in the presence of sulindac and dacarbazine.

Drug	Concentration [µM]	Average CAT activity		
		[nmol/min/ml] ± SD	[nmol/min/mg protein] ± SD	[%]
Sulindac	control	9.28 ± 0.56	4.12 ± 0.25	100.0 ± 6.0
	0.1	9.03 ± 0.57	3.99 ± 0.26	99.8 ± 6.3
	1.0	8.04 ± 0.46	3.54 ± 0.21	87.8 ± 5.1
	10	7.82 ± 0.61	3.45 ± 0.27	83.8 ± 6.6
	50	7.36 ± 0.40	3.31 ± 0.18	80.4 ± 4.4
	100	5.85 ± 0.50	2.57 ± 0.22	62.4 ± 5.3
Dacarbazine	control	9.28 ± 0.56	4.12 ± 0.25	100.0 ± 6.0
	0.1	8.74 ± 0.48	3.75 ± 0.21	94.7 ± 5.2
	1.0	8.32 ± 0.39	3.80 ± 0.18	92.4 ± 4.4
	10	8.14 ± 0.38	3.64 ± 0.17	88.5 ± 4.2
	50	7.45 ± 0.42	3.38 ± 0.19	82.0 ± 4.6
	100	7.39 ± 0.38	3.28 ± 0.17	79.7 ± 4.1

Table 4S. Catalase activity in C32 amelanotic melanoma cells cultured in the presence of sulindac and dacarbazine.

Drug	Concentration [µM]	Average CAT activity		
		[nmol/min/ml] ± SD	[nmol/min/mg protein] ± SD	[%]
Sulindac	control	1.13 ± 0.09	0.59 ± 0.05	100.0 ± 8.1
	10	1.11 ± 0.09	0.57 ± 0.05	98.0 ± 8.1
	50	1.11 ± 0.09	0.57 ± 0.05	98.2 ± 8.1
	100	1.15 ± 0.07	0.59 ± 0.03	101.6 ± 5.7
Dacarbazine	control	1.13 ± 0.09	0.59 ± 0.05	100.0 ± 8.1
	10	1.03 ± 0.07	0.53 ± 0.03	90.8 ± 5.7
	50	0.97 ± 0.07	0.50 ± 0.03	85.3 ± 5.7
	100	0.89 ± 0.09	0.46 ± 0.05	78.1 ± 8.1

Table 5S. Glutathione peroxidase activity in COLO829 melanotic melanoma cells cultured in the presence of sulindac and dacarbazine.

Drug	Concentration [µM]	Average GPx activity		
		[nmol/min/ml] ± SD	[nmol/min/mg protein] ± SD	[%]
Sulindac	control	9.82 ± 0.43	4.36 ± 0.19	100.0 ± 4.4
	0.1	9.54 ± 0.50	4.34 ± 0.23	99.4 ± 5.2
	1.0	9.12 ± 0.49	4.11 ± 0.22	94.1 ± 5.1
	10	8.74 ± 0.51	3.85 ± 0.23	88.2 ± 5.2
	50	7.67 ± 0.45	3.44 ± 0.20	78.8 ± 4.6
	100	7.21 ± 0.45	3.16 ± 0.20	72.5 ± 4.5
Dacarbazine	control	9.82 ± 0.43	4.36 ± 0.19	100.0 ± 4.4
	0.1	9.38 ± 0.37	4.19 ± 0.16	96.1 ± 3.8
	1.0	8.80 ± 0.30	4.02 ± 0.14	92.3 ± 3.1
	10	8.15 ± 0.59	3.65 ± 0.26	83.7 ± 6.0
	50	7.33 ± 0.45	3.32 ± 0.21	76.2 ± 4.7
	100	7.29 ± 0.40	3.24 ± 0.18	74.3 ± 4.0

Table 6S. Glutathione peroxidase activity in C32 amelanotic melanoma cells cultured in the presence of sulindac and dacarbazine.

Drug	Concentration [µM]	Average GPx activity		
		[nmol/min/ml] ± SD	[nmol/min/mg protein] ± SD	[%]
Sulindac	control	26.06 ± 1.04	13.45 ± 0.53	100.0 ± 4.0
	10	26.09 ± 1.57	13.45 ± 0.81	100.0 ± 6.0
	50	25.33 ± 1.86	13.08 ± 0.96	97.2 ± 7.1
	100	24.84 ± 1.56	12.79 ± 0.80	95.1 ± 6.0
Dacarbazine	control	26.06 ± 1.04	13.45 ± 0.53	100.0 ± 4.0
	10	24.40 ± 0.64	12.58 ± 0.33	93.5 ± 2.4
	50	23.46 ± 0.63	12.10 ± 0.33	89.9 ± 2.4
	100	22.16 ± 1.37	11.45 ± 0.71	85.1 ± 5.3

Table 7S. Content of hydrogen peroxide in COLO829 melanotic melanoma cells cultured in the presence of sulindac and dacarbazine.

Drug	Concentration [µM]	Content of H_2O_2		
		[µmol/ml] ± SD	[µmol/mg protein] ±SD	[%]
Sulindac	control	47.12 ± 1.51	20.24 ± 0.65	100.0 ± 3.2
	0.1	49.94 ± 1.32	20.90 ± 0.55	103.2 ± 2.7
	1.0	49.28 ± 1.91	21.35 ± 0.83	105.4 ± 4.1
	10	52.96 ± 2.19	22.50 ± 0.93	111.1 ± 4.6
	50	63.90 ± 2.50	26.46 ± 1.05	130.7 ± 5.2
	100	70.42 ± 3.06	29.82 ± 1.30	147.3 ± 6.4
Dacarbazine	control	47.12 ± 1.51	20.24 ± 0.65	100.0 ± 3.2
	0.1	58.35 ± 2.98	25.50 ± 1.30	123.9 ± 6.3
	1.0	61.38 ± 2.51	27.22 ± 1.11	132.2 ± 5.4
	10	68.60 ± 3.66	30.53 ± 1.63	148.3 ± 7.9
	50	72.38 ± 2.36	32.23 ± 1.05	156.6 ± 5.1
	100	77.42 ± 2.88	33.87 ± 1.26	164.6 ± 6.1

Table 8S. Content of hydrogen peroxide in C32 amelanotic melanoma cells cultured in the presence of sulindac and dacarbazine.

Drug	Concentration [µM]	Content of H_2O_2		
		[µmol/ml] ± SD	[µmol/mg protein] ±SD	[%]
Sulindac	control	639.15 ± 22.20	132.00 ± 4.58	100.0 ± 3.5
	10	653.43 ± 14.86	134.69 ± 3.06	102.0 ± 2.3
	50	639.25 ± 30.48	132.00 ± 6.29	100.0 ± 4.8
	100	651.39 ± 21.02	134.21 ± 4.33	101.7 ± 3.3
Dacarbazine	control	639.15 ± 22.20	132.00 ± 4.58	100.0 ± 3.5
	10	769.44 ± 22.00	158.73 ± 4.54	120.3 ± 3.4
	50	816.55 ± 21.41	168.48 ± 4.42	127.6 ± 3.3
	100	854.54 ± 27.59	176.53 ± 5.70	133.7 ± 4.3

Table 9S. Content of protein p53 in COLO829 melanotic melanoma cells cultured in the presence of sulindac and dacarbazine.

Drug	Concentration [µM]	Content of p53	
		[U/mg protein] ±SD	[%]
Sulindac	control	2.74 ± 0.17	100.0 ± 6.2
	0.1	3.25 ± 0.15	118.7 ± 5.5
	1.0	3.30 ± 0.15	120.5 ± 5.4
	10	3.52 ± 0.22	128.5 ± 7.9
	50	4.20 ± 0.23	153.4 ± 8.6
	100	5.43 ± 0.40	198.1 ± 4.7
Dacarbazine	control	2.74 ± 0.17	100.0 ± 6.2
	0.1	3.11 ± 0.10	113.4 ± 3.7
	1.0	3.38 ± 0.26	123.4 ± 9.3
	10	3.64 ± 0.24	132.8 ± 8.8
	50	4.28 ± 0.25	156.3 ± 9.2
	100	5.12 ± 0.31	186.7 ± 11.3

Table 10S. Content of protein p53 in C32 amelanotic melanoma cells cultured in the presence of sulindac and dacarbazine.

Drug	Concentration [μM]	Content of p53	
		[U/mg protein] ±SD	[%]
Sulindac	control	6.35 ± 0.19	100.0 ± 3.0
	10	6.37 ± 0.29	100.3 ± 4.5
	50	6.60 ± 0.58	104.0 ± 9.2
	100	8.01 ± 0.34	126.1 ± 5.4
Dacarbazine	control	6.35 ± 0.19	100.0 ± 3.0
	10	6.46 ± 0.34	101.8 ± 5.4
	50	6.83 ± 0.33	107.6 ± 5.2
	100	8.59 ± 0.51	135.3 ± 8.0

Table 11S. Content of protein Bcl-2 in COLO829 melanotic melanoma cells cultured in the presence of sulindac and dacarbazine.

Drug	Concentration [μM]	Content of Bcl-2	
		[ng/mg protein] ±SD	[%]
Sulindac	control	10.15 ± 0.75	100.0 ± 7.4
	0.1	9.62 ± 0.63	94.7 ± 6.2
	1.0	8.47 ± 0.38	83.4 ± 3.7
	10	7.36 ± 0.72	72.5 ± 7.1
	50	7.00 ± 0.81	68.9 ± 8.0
	100	6.81 ± 0.42	67.1 ± 4.2
Dacarbazine	control	10.15 ± 0.75	100.0 ± 7.4
	0.1	8.38 ± 0.68	82.5 ± 6.7
	1.0	7.21 ± 0.48	71.0 ± 4.7
	10	4.11 ± 0.57	40.5 ± 5.6
	50	3.52 ± 0.51	34.7 ± 5.1
	100	3.33 ± 0.33	32.8 ± 3.3

Table 12S. Content of protein Bcl-2 in C32 amelanotic melanoma cells cultured in the presence of sulindac and dacarbazine.

Drug	Concentration [µM]	Content of Bcl-2	
		[ng/mg protein] ±SD	[%]
Sulindac	control	3.57 ± 0.13	100.0 ± 3.8
	10	4.94 ± 0.18	138.5 ± 5.0
	50	5.64 ± 0.32	158.2 ± 9.1
	100	7.84 ± 0.28	219.9 ± 7.7
Dacarbazine	control	3.57 ± 0.13	100.0 ± 3.8
	10	5.30 ± 0.18	148.5 ± 5.0
	50	5.54 ± 0.22	155.3 ± 6.1
	100	6.00 ± 0.22	168.2 ± 6.3

Table 13S. Content of protein Bax in COLO829 melanotic melanoma cells cultured in the presence of sulindac and dacarbazine.

Drug	Concentration [µM]	Content of Bax	
		[pg/mg protein] ±SD	[%]
Sulindac	control	393.24 ± 12.87	100.0 ± 3.3
	0.1	397.55 ± 8.76	101.1 ± 2.2
	1.0	402.01 ± 12.48	102.2 ± 3.2
	10	410.27 ± 15.96	104.3 ± 4.1
	50	411.26 ± 10.40	104.6 ± 2.6
	100	431.48 ± 16.21	109.7 ± 4.1
Dacarbazine	control	393.24 ± 12.87	100.0 ± 3.3
	0.1	402.81 ± 12.71	102.4 ± 3.2
	1.0	403.92 ± 17.13	102.7 ± 4.4
	10	410.27 ± 18.74	104.3 ± 4.8
	50	417.17 ± 10.83	106.1 ± 2.8
	100	443.31 ± 13.41	112.7 ± 3.4

Table 14S. Content of protein Bax in C32 amelanotic melanoma cells cultured in the presence of sulindac and dacarbazine.

Drug	Concentration [µM]	Content of Bax	
		[pg/mg protein] ±SD	[%]
Sulindac	control	465.01 ± 14.90	100.0 ± 3.2
	10	468.95 ± 19.79	100.8 ± 4.3
	50	466.16 ± 15.84	100.2 ± 3.4
	100	496.58 ± 26.40	106.8 ± 5.7
Dacarbazine	control	465.01 ± 14.90	100.0 ± 3.2
	10	470.89 ± 14.38	101.3 ± 3.1
	50	497.00 ± 15.76	106.9 ± 3.4
	100	502.37 ± 16.53	108.0 ± 3.6
